# Density functional theory study of palladium cluster adsorption on a graphene support

**DOI:** 10.1039/d0ra01059f

**Published:** 2020-05-29

**Authors:** Riaz Hussain, Muhammad Saeed, Muhammad Yasir Mehboob, Saif Ullah Khan, Muhammad Usman Khan, Muhammad Adnan, Mahmood Ahmed, Javed Iqbal, Khurshid Ayub

**Affiliations:** Department of Chemistry, University of Okara Okara 56300 Pakistan; Department of Chemistry, Government College University Faisalabad 38000 Pakistan; College of Natural Sciences, Department of Chemistry, Chosun University South Korea; Renacon Pharma Limited Lahore Pakistan; Department of Chemistry, University of Agriculture Faisalabad 38000 Pakistan Javedkhattak79@gmail.com; Department of Chemistry, COMSATS Institute of Information Technology Abbottabad 22060 Pakistan Khurshid@cuiatd.edu.pk

## Abstract

The geometric, thermodynamic and electronic properties of Pd–graphene nanocomposites are comprehensively studied through quantum mechanical methods. Geometries of these clusters are optimized with the well-calibrated Minnesota functional M06-2X. The adsorption energies calculated at the M06-2X/LANL2DZ level show better agreement with those calculated from MP2/ANO-RCC-VDZP. Two different representative models for graphene, coronene and hexabenzocoronene, are used. The adsorption energies analysis reveals that the interaction energies increase with the size of the adsorbed cluster. However, for Pd_*n*_/hexabenzocoronene, the interaction energies show a sudden drop at Pd_8_/hexabenzocoronene. The difference in behavior between the interaction energies of Pd_*n*_/hexabenzocoronene and Pd_*n*_/coronene is attributed to the edge effect present in coronene. The electronic properties, including highest occupied molecular orbital (HOMO) and lowest unoccupied molecular orbital (LUMO), Fermi level, molecular electrostatic potential (MEP), dipole moment, vertical ionization potential (VIP), vertical electron affinity (VEA), chemical hardness (*η*), softness (*S*) and chemical potential (*μ*) are studied. The VIP and VEA reveal that Pd_*n*_/coronene clusters are stable in nature with the least reactivity. The HOMO–LUMO energy gaps are reduced with the increase in cluster size. The electronic properties show irregular trends, where the most favorable electronic properties are obtained for Pd_7_/coronene and Pd_10_/coronene.

## Abstract 2

The geometric, thermodynamic and electronic properties of Pd–graphene nanocomposites are comprehensively studied through quantum mechanical methods.

## Graphical abstract

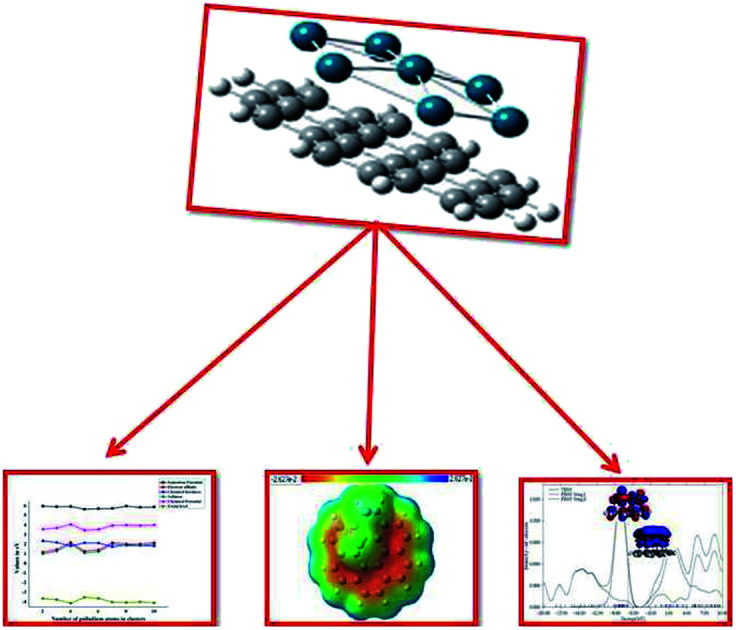

## 1. Introduction

Metal clusters are considered as intermediates between solid states, and these molecules generally exhibit unexpected physical properties owing to the quantum size effect.^1^ The properties and structure of atomic clusters have attracted the interest of many theoreticians with the quick advancement of computer technology.^2–4^ The properties of clusters rely upon the size and composition of the system, and these are distinct from the bulk due to their specific properties. Clusters comprising transition metal atoms such as platinum (Pt) and palladium (Pd) are of immense significance due to their applications in heterogeneous catalysis.^5^ Palladium is a rare, lustrous silvery white metal which belongs to group 10 of the modern periodic table. Pd clusters are utilized in the exhaust system of automobiles in order to control the emissions of toxic pollutants such as CO, NO and hydrocarbons from vehicles. Finely dispersed clusters of palladium in alumina are more efficient surfaces for the oxidation of CO than Pd (111) single crystals.^6^ Trace quantities of methane gas in the atmosphere (in the range of 6–10 ppm) can be detected by Pd metal clusters; hence, these metal clusters are efficient sensors for methane detection. A practical sensor for the detection of methane involves loading of Pd clusters on single-walled carbon nanotubes (SWNTs).^7^

Theoretical calculations are efficient tools for studying the various electronic and structural properties of the transition metal clusters.^8–11^ Many reports in the literature illustrate the structure and properties of Pd clusters. For example, density functional theory on neutral, ligand-free palladium clusters (*n* = 2–309) was described, and it suggested that the clusters Pd_4–7,9,13_ are the most stable clusters.^12^ Another report on ligand stabilized-palladium clusters suggested that palladium, as metal core, exhibited exceptional structural features in these clusters.^13^ Similarly, the magnetic behaviour of neutral and anionic Pd_*n*_ is reported in the literature. The magnetic properties of palladium clusters depend upon temperature. For example, the magnetic moment for Pd_7_ was enhanced as temperature was increased.^14^ Further, energy states of small Pd clusters have also been investigated in order to understand the working mechanism of metallic nanoprobes.^15^

The deposition of metal on many surfaces has been broadly investigated for the understanding of surface phenomena and catalysis. Most of the time, carbonaceous materials, zeolites or oxides are utilized as support or adsorbent.^16^ A carbon-based support is utilized due to its electrical conductivity and inertness of the surface.^17–20^ A well-known carbonaceous material used as support for palladium metal clusters is graphene. Graphene is an unlimited two-dimensional material; therefore, small models are generally used for theoretical calculations.^21–23^ Coronene is a polycyclic aromatic hydrocarbon with the molecular formula C_24_H_12_,^24^ which is generally used as a model for graphene.

Granatier *et al.* investigated the interaction of graphene with dimers and tetramers of silver, gold and palladium with MP2 and DFT methods. The validity of the MP2 calculations was confirmed for benzene–metal dimer complexes with benchmark values obtained at the CCSD(T) level. They used the coronene molecule as a representative model for graphene structure. It was found that silver and gold clusters bind with carbon surface through dispersion and charge transfer interactions, whereas the palladium clusters bind with carbon surface through dative bonds. Calculations on coronene metal complexes at MP2 level indicated that the binding energies of palladium, silver and gold tetramers are higher than those of the corresponding dimers. Among DFT methods, results from M06-2X methods are comparable to those from MP2 calculations. Moreover, the binding energies of coronene metal complexes in water at the M06-2X level are only slightly lower than those in vacuum. These investigations were also supported by the results of stability of metal nanoparticles (∼20 nm) on graphene composites observed by scanning electron microscopy (SEM).^25–27^

In another combined experimental and theoretical report, Rao and coworkers studied the metal nanoparticle-decorated graphene structures. They examined the interaction between graphene and metal nanoparticles of Ag, Au, Pt and Pd by employing Raman spectroscopy and compared their results with theoretical calculations, but this study was limited to only the M40 cluster on graphene. Similarly, the interaction of graphene with other metals such as cobalt is also limited to only four atoms. The studies of Granatier and Rao regarding the interaction mechanism between metal cluster and graphene are quite important (although different models are used); however, they are limited to only 2, 4 or 40 atoms of metal cluster on graphene. Further, Pd adsorption on graphene was examined in past, which suggested that Pd atoms deposited on graphene surface had a great tendency for cluster formation.^28^ Clusters of palladium (Pd_1–6_) supported on graphene^29^ were studied for H_2_ adsorption. Similarly, the stability of different Pd clusters supported on pristine, B-doped, and defective graphene as well as their reactivity towards oxygen was successfully reported in valuable literature.^30^

The interaction energies, geometries and electronic properties of medium-sized clusters on graphene surface are not reported in the literature. With this motivation, we studied here the detailed theoretical analysis of geometries, binding energies and electronic properties of small palladium clusters on coronene, a model for graphene.^31^ Moreover, the most stable orientations of Pd_*n*_ clusters were also studied on hexabenzocoronene. This is the first study of its type where a complete range of palladium clusters Pd_*n*_ (*n* = 2–10) is studied on the surface of coronene.

## 2. Computational procedure

All calculations are performed with Gaussian 09. The M06-2X function with Los Alamos LANL2DZ effective core potentials is used for optimization of the Pd_*n*_/coronene (*n* = 2–10) system. A number of different orientations of palladium clusters on coronene are considered in order to find the lowest energy structure on the potential energy surface. The most stable geometries of palladium clusters for adsorption on coronene are taken from the literature.^32^ These palladium clusters are adsorbed on the coronene (in the present study) and allowed to fully relax during optimization without any constraints. Optimization of palladium coronene composite is carried out at different spin states in order to find the lowest energy spin state for each complex. The spin multiplicity of Pd_*n*_/coronene composites is shown in Table 2. The optimized geometries are confirmed as true minima on potential energy surface through vibrational analysis. Lack of any imaginary frequencies confirmed the true minima nature of these complexes.

The adsorption energy of the clusters with coronene is calculated using the following equation:

1 *E*_ad_ = *E*_cluster–coronene_ − (*E*_cluster_ + *E*_coronene_),

where *E*_cluster–coronene_, *E*_cluster_ and *E*_coronene_ are the total energies of the Pd_*n*_/coronene (*n* = 2–10), Pd_*n*_ clusters and isolated coronene, respectively.^33^

Chemical hardness (*η*) is the parameter used to determine the chemical reactivity of the system. In computational terms (DFT), at constant potential, it is the double derivative of energy with respect to the total number of electrons. Furthermore, corresponding stabilities of systems and bulk are indicated through the application of the principle of maximum hardness (PMH) given by Pearson.^34^2
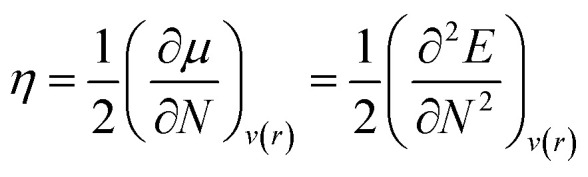
where *η* is the chemical hardness of the system, *N* is the number of electrons, and *E* is energy of the system. External potential is mentioned as *v*(*r*), and *μ* is the chemical potential that leads to hardness. With the help of VIP and VEA, the global hardness can be measured through Koopmans' theorem.^35–37^ The equation of global hardness is shown as:3
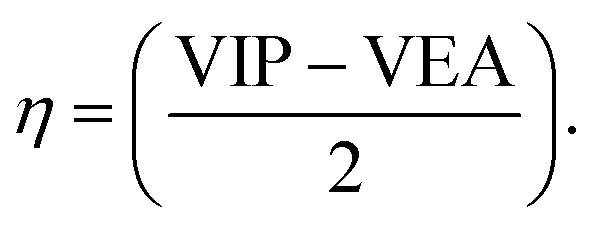


VIP and VEA are the vertical ionization potential and electron affinity, respectively. The inverse of hardness is known as softness of the system.^38^ The mathematical equation is as follows:4
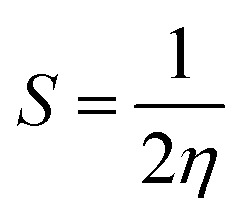


It is defined through DFT that electron density *ρ*(*r*) is the measurement of the energy of the molecules or atoms in a ground state,^39^ and can be written as:

5 *E*[*ρ*] = *F*_HK_[*ρ*] + ∫*v*(*r*)*ρ*(*r*)d*r*,

where *v*(*r*) represents the external potential, and *F*_HK_ is the universal Hohenberg–Kohn function, which is obtained by adding the interaction energy of electron–electron (*V*_∞_[*ρ*]) and electronic kinetic energy (T[*ρ*]).

6 *F*_HK_[*ρ*] = *T*[*ρ*] + *V*_ee_[*ρ*]

when the first partial derivative of *E*[*ρ*] is taken by keeping the external potential [*v*(*r*)] constant with regards to the number of electrons (*N*), which then this gives the chemical potential (*μ*) as follows:^40^

7 d*E* = *μ*d*N* + ∫*ρ*(*r*)d*v*(*r*)d*r*

8
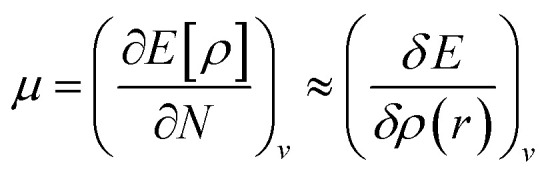


By using the finite method and the curvature of *E* with respect to the number of electrons (*N*) at the proposed value of *N* at 0 K, the chemical potential (*μ*) can be written as:^41^9
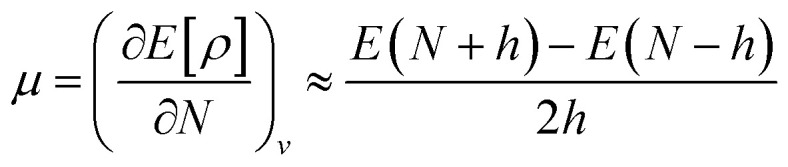


VIP and VEA information gives knowledge about the integral value of *N*; the infinitesimally small quantity h can be set equal to unity, and through rearrangement, followed by using electron densities of *N*_0_, *N*_0_ − 1, and *N*_0_ + 1 electron systems, the equation is modified as:10
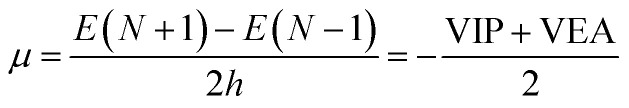


From the understanding of MO theory, within the framework of density functional theory, Koopmans' approximation is applied to Kohn Sham orbitals, through which the above equation can be enhanced by using the MO energy values (*ε*) of the highest occupied (*E*_HOMO_) and lowest occupied (*E*_LUMO_) molecular orbitals as:11
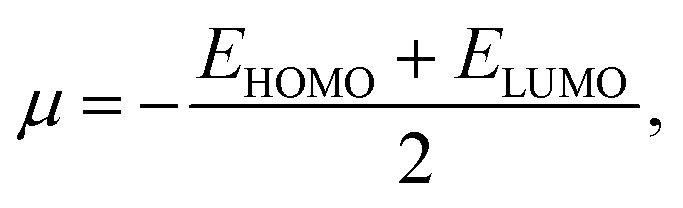
where *μ* is the chemical potential, *E*_HOMO_ is the energy of the highest occupied molecular orbital and *E*_LUMO_ is the energy of the lowest unoccupied molecular orbital.

The HOMO–LUMO gap is a measure of electron jump from the occupied orbitals to unoccupied orbitals, and it reflects the electronic stability of the Pd_*n*_/coronene (*n* = 2–10) system. The following equation is used to calculate the gap between HOMO and LUMO:^42,43^

12 *E*_g_ = *E*_LUMO_ − *E*_HOMO_

Advancement in electronic data of our system is indicated through the Fermi level, which is presented by *E*_FL_.^44–48^ The following equation is used to acquire *E*_FL_:13
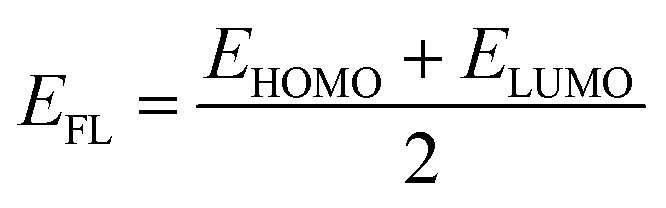


The VIP and VEA of the system is obtained from (−*E*_HOMO_) and (−*E*_LUMO_), respectively.

## 3. Results and discussion

### 3.1 Adsorption energies and geometries of Pd_*n*_/coronene (*n* = 2–10) composites

The M06-2X functional of DFT is chosen for palladium graphene clusters because it is a validated method for the Pd–graphene cluster based on comparison of the results with those of MP2 and CCSD(T) methods. In our study, initially, two different basis sets are utilized to calculate the binding energies of the Pd_2_/coronene in order to realize any improvement in the calculation of interaction energy (from those available in the literature). In one approach, a mixed basis set is applied, in which palladium atoms are treated with LanL2DZ pseudopotential, whereas all carbon and hydrogen atoms are treated with 6-31G (d,p) Pople-type basis set, as shown in Table 1.

**Table 1:** The binding energies (kcal mol^−1^) of Pd_2_ and Pd_4_ clusters over coronene substrate calculated at ANO-RCC-VDZP, LANL2DZ and mixed basis set [6-31G (d,p) & LANL2DZ]

Systems	Methods	ANO-RCC-VDZP	6-31G (d, p) & LANL2DZ	LANL2DZ
Pd_2_/coronene	MP2	31.22	—	—
Pd_2_/coronene	M06-2X	23.68	18	30
Pd_4_/coronene	MP2	39.03	—	—
Pd_4_/coronene	M06-2X	45.46	29.05	36.71

In the other approach, LANL2DZ basis set is used for all atoms. Unfortunately, the values of the adsorption energies obtained from the mixed basis set are no better than the previously reported values. In the literature, adsorption energy values obtained for Pd_4_/coronene at MP2/ANO-RCC-VDZP and M06-2X/ANO-RCC-VDZP are 39.03 and 45.46 kcal mol^−1^, respectively,^49^ and the value obtained for Pd_4_/coronene from the mixed basis set is 29.05 kcal mol^−1^, which is about 9.98 and 16.41 kcal mol^−1^ lower than the reported values at MP2/ANO-RCC-VDZP and M06-2X/ANO-RCC-VDZP level, respectively. The value obtained from M06-2X/LANL2DZ is 36.71 kcal mol^−1^, which shows best agreement with the reported values.

**Fig. 1:**
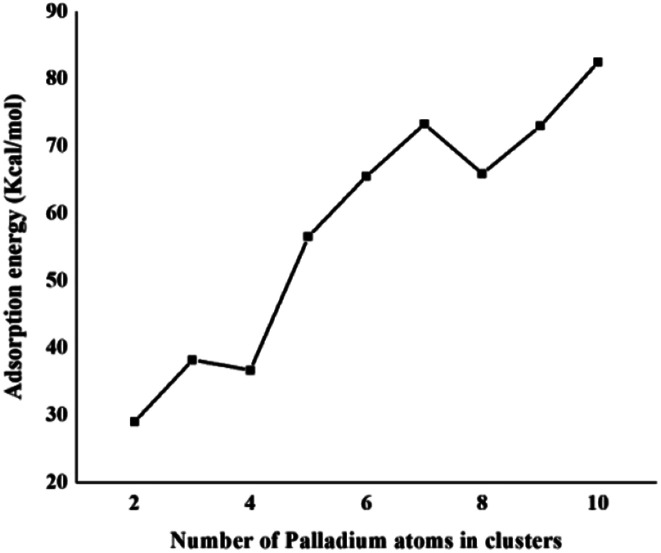
Binding energy (kcal mol^−1^) of the most stable Pd_*n*_/coronene (*n* = 2–10) composites.

The same trend is also observed for Pd_2_/coronene. The reported binding energies for Pd_2_/coronene at MP2/ANO-RCC-VDZP and M06-2X/ANO-RCC-VDZP are 31.22 kcal mol^−1^ and 23.68 kcal mol^−1^, respectively. The binding energy obtained from the mixed basis set for Pd_2_/coronene is 18 kcal mol^−1^, which is about 5.68 and 5.37 kcal mol^−1^ lower than the reported values at MP2/ANO-RCC-VDZP and M06-2X/ANO-RCC-VDZP level, respectively. Binding energy obtained from the M06-2X/LanL2DZ is 30 kcal mol^−1^, which is closer to the reported value at MP2/ANO-RCC-VDZP. Moreover, the binding energies calculated at M06-2X/LANL2DZ are in better agreement with those from MP2 methods than those available in the literature at M06-2X/ANO-RCC-VDZP. It is clear from our calculations that the mixed set is not suitable for study; therefore, density functional theory study at the best-chosen level (M06-2X/LANL2DZ) is performed for the structural, electronic and thermodynamic properties of Pd_*n*_/coronene (*n* = 2–10) composites. The clusters exist in different shapes for each cluster size. The most stable palladium clusters were chosen for the calculation of palladium cluster adsorption on coronene (see Computational methodology). It is also clear from Fig. 1 that the increase of adsorption energy with the increase in number of the palladium atoms in clusters is not steady. The highest value of binding energy is shown by the Pd_10_/coronene, which is 82.46 kcal mol^−1^, whereas the lowest adsorption energy is for Pd_2_/coronene (*E*_d_ = 29.05 kcal mol^−1^). Adsorption energies of Pd_7_/coronene and Pd_9_/coronene are quite comparable to each other, with 73.00 and 73.31 kcal mol^−1^ for Pd_7_/coronene and Pd_9_/coronene, respectively. However, Pd_4_/coronene reveals the second lowest binding energy (*E*_d_ = 36.71 kcal mol^−1^) in our study of Pd_*n*_/coronene (*n* = 2–10) composites. For example, Pd_10_/coronene is the system with the maximum number of palladium atoms on the coronene (as shown in Fig. 3), resulting in the highest adsorption energy (highest stability). Similarly, Pd_7_/coronene has almost seven palladium atoms interacting with the coronene, which results in the 2^nd^ highest adsorption energy. In the case of Pd_9_/coronene, five atoms interact with the coronene, which gives the 3^rd^ highest binding energy (*E*_d_ = 73.00 kcal mol^−1^). However, in case of the Pd_6_/coronene (*E*_d_ = 65.56 kcal mol^−1^) and Pd_8_/coronene (*E*_d_ = 65.91 kcal mol^−1^) systems, minute difference in adsorption energy is seen despite different numbers of interacting atoms. This minute difference in adsorption energy might be due to the difference in distance of palladium clusters from coronene in both cases.

**Fig. 2:**
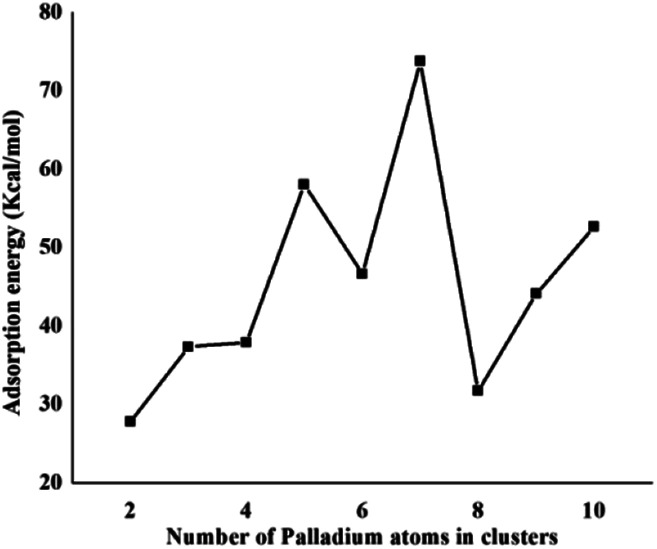
Binding energy (kcal mol^−1^) of the most stable Pd_*n*_/HBC (*n* = 6–10) composites.

Coronene is a smaller model for examining the interaction energies of graphene with palladium clusters. It is expected that with increase in the size of the cluster, the chances of palladium atoms interacting with the edges of coronene increase. The edges can cause unusual increase in the interaction energies due to edge effect. Therefore, we have optimized most stable palladium clusters on hexabenzocoronene (HBC) in order to eliminate the edge effect. The interaction mechanism of palladium cluster–decorated hexabenzocoronene is investigated with the M062X method of DFT. The most stable orientations of Pd_*n*_ on coronene are taken as model for the Pd_*n*_/HBC complexes. Pd_2_/HBC, Pd_3_/HBC, Pd_4_/HBC and Pd_7_/HBC systems are stable in singlet spin state, whereas Pd_5_/HBC, Pd_6_/HBC, Pd_8_/HBC, Pd_9_/HBC and Pd_10_/HBC systems are stable in triplet spin state. From Fig. 2, it is clearly noted that the increase in adsorption energy of Pd_*n*_/HBC systems with increase in cluster size is not uniform. The smallest adsorption energy value is seen in the case of Pd_2_/HBC system (*E*_ad_ = 27.81 kcal mol^−1^), which might be due to a lower number of palladium atoms interacting with HBC, and the largest adsorption energy value is noted in the case of Pd_7_/HBC system (*E*_ad_ = 73.76 kcal mol^−1^), and this is due to planar configuration of the palladium cluster over HBC (see Fig. 4). Pd_3_/HBC (37.34 kcal mol^−1^) and Pd_4_/HBC (37.89 kcal mol^−1^) have approximately similar values of adsorption energy with minute difference. A sudden drop in adsorption energy is seen in the case of Pd_8_/HBC system (*E*_ad_ = 31.76 kcal mol^−1^), and this might be due to the smaller number of palladium atoms present in the lower-lying plane of a cluster that interacts directly with the HBC ring. Moreover, the interaction energies of Pd_6_/HBC and Pd_9_/HBC are smaller than that of Pd_10_/HBC, which strongly suggests that the edge effect strongly prevailed in the corresponding coronene complexes.

**Fig. 3:**
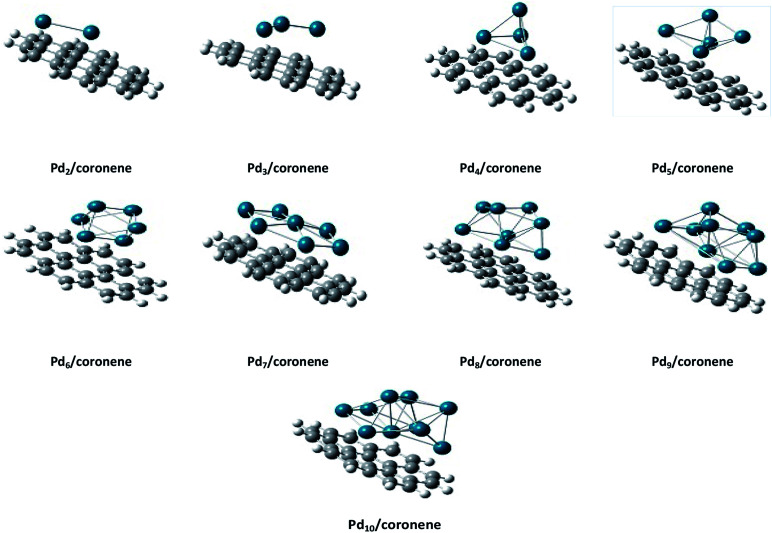
Side view of the most stable Pd_*n*_/coronene (*n* = 2–10) composite systems.

**Fig. 4:**
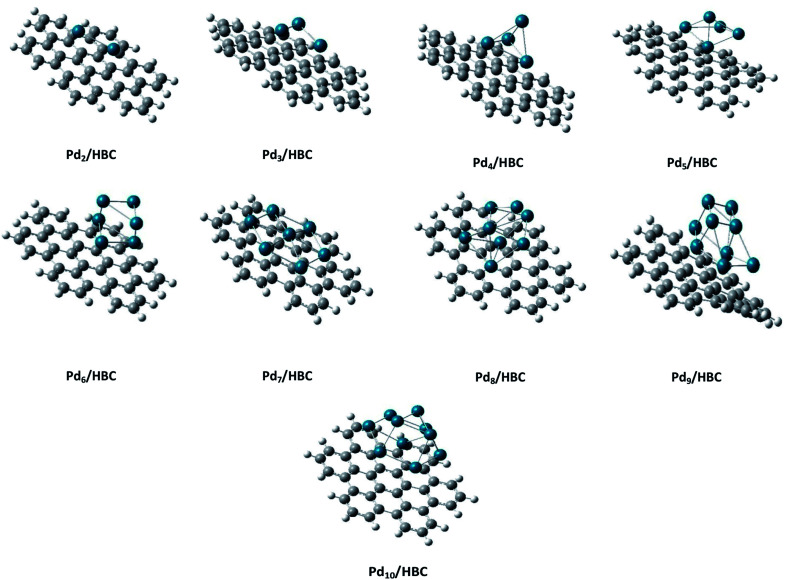
Side view of the most stable Pd_*n*_/HBC (*n* = 2–10) composite systems.

### 3.2 Frontier molecular orbital analysis (FMO) of the most stable Pd_*n*_/coronene (*n* = 2–10) composites

**Fig. 5:**
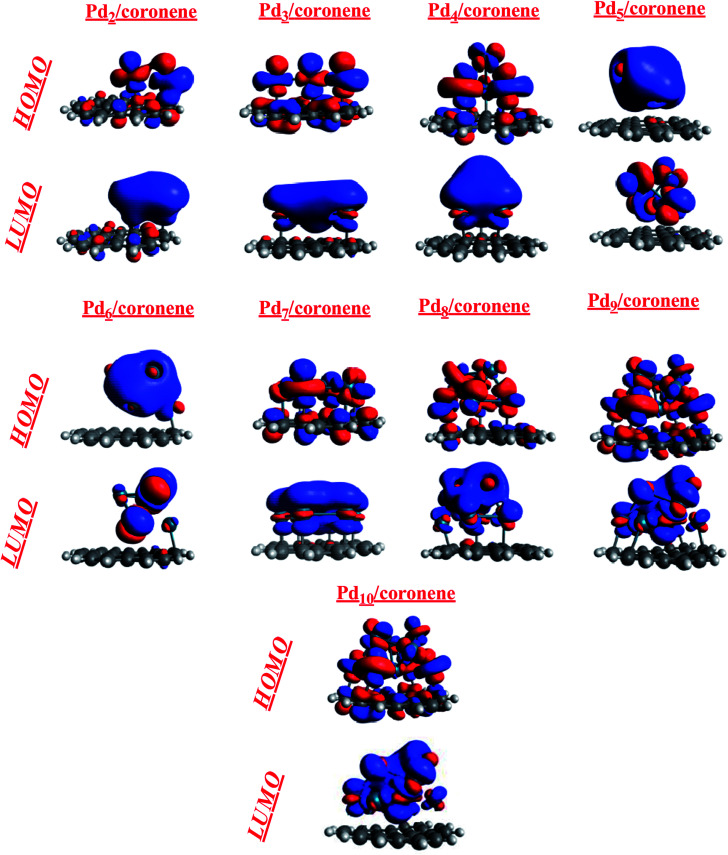
HOMO–LUMO orbitals (side view) of Pd_*n*_/coronene (*n* = 2–10) composite systems.

The theory of frontiers molecular orbital (FMO) was used to describe the interaction of the palladium clusters with coronene. The HOMO and LUMO are related to the frontier molecular orbital analysis (Fig. 5). The HOMO and LUMO energies of coronene are −6.79 and −0.90 eV, respectively, with an energy gap of 5.88 eV. Slight increase in HOMO energies and significant decrease in LUMO energies was noted when palladium atoms are adsorbed on coronene (Table 2). Among Pd_*n*_/coronene clusters, the highest HOMO–LUMO gap (4.80 eV) is observed for Pd_2_/coronene, where the energies of HOMO and LUMO are −6.01 and −1.21 eV, respectively. The lowest value of HOMO is for Pd_5_/coronene (−5.69 eV), which resulted in a narrow band gap with a value of 4.38 eV. As the difference between the energies of the highest occupied molecular orbital (HOMO) and the lowest unoccupied molecular orbital (LUMO) increases, the chances of electrons going to LUMO from HOMO decrease.^50^ The highest value of the H–L gap for Pd_2_/coronene illustrates its higher kinetic stability because HOMO–LUMO gap is the indication of the kinetic stability of composites.^51^ It is obvious from Table 2 that the HOMO–LUMO gap decreases with increase in the number of palladium atoms on the coronene up to Pd_4_/coronene, beyond which there is a mixed trend of HOMO–LUMO gap. One may argue that the HOMO–LUMO gap becomes lower and lower when the number of palladium atoms interacting with the coronene increases to a maximum of four atoms (Pd_4_/coronene) on coronene. In our system, from Pd_5_/coronene to Pd_10_/coronene, there is a mixed trend, *i.e.*, Pd_7_/coronene has the lowest HOMO–LUMO energy gap, whereas Pd_10_/coronene shows a narrower energy gap than Pd_8_/coronene, Pd_9_/coronene, Pd_6_/coronene and Pd_5_/coronene. These results illustrate that the difference in HOMO–LUMO energy gap is due to different numbers of metal atoms interacting with coronene due to the geometry and angle of adsorption on the coronene surface, as shown in Fig. 3. The HOMO–LUMO gap depends on the number of the interacting atoms with coronene, and the systems with the large number of interacting atoms have low HOMO–LUMO gaps. For example, Pd_5_/coronene, Pd_6_/coronene, Pd_7_/coronene, Pd_8_/coronene, Pd_9_/coronene and Pd_10_/coronene have 5, 6, 7, 8, 9 and 10 palladium atoms interacting with coronene, respectively, and they have low values of HOMO–LUMO energy gap as compared to coronene. Pd_2_/coronene, Pd_3_/coronene and Pd_4_/coronene have 2, 3 and 4 palladium atoms interacting with coronene, and their HOMO–LUMO gaps decrease gradually. For better understanding of the adsorption process of Pd clusters on coronene, densities of HOMO and LUMO are analyzed. Pd_*n*_/coronene (*n* = 2–10) systems are divided into different categories depending on the distribution of the HOMO and LUMO densities on the metal cluster and coronene. In the first category, the density of highest occupied molecular orbitals (HOMO) and lowest unoccupied molecular orbitals (LUMO) are similar; HOMO and LUMO are equally located on metal clusters and coronene, and this situation is shown by Pd_2_/coronene only. This may be attributed to orbital energy match between coronene and the metal cluster. The second category includes the systems in which HOMO is equally distributed on both palladium clusters and coronene, but the LUMO density is congested on the cluster. Such systems include Pd_3_/coronene, Pd_4_/coronene, Pd_7_/coronene and Pd_10_/coronene. It is due to the high energy of HOMO. Therefore, it may be argued that the cluster where the HOMO is present on both fragments and the LUMO density is located only on the palladium cluster leads to lower HOMO–LUMO gap, except Pd_3_/coronene. The third category includes such systems in which HOMO and LUMO show the density only on the metal clusters, and systems having such situation are Pd_5_/coronene, Pd_6_/coronene and Pd_9_/coronene; this situation results from the shifting of electron density on the metal clusters. This clearly illustrates that HOMO energy levels of the transition metal (Pd) are higher in energy than coronene. When combined, the palladium atoms lead to generation of new states between the original HOMO and LUMO of coronene.

**Table 2:** The spin multiplicity, vertical ionization potential (eV), vertical electron affinity (eV), energies of highest occupied molecular orbitals (HOMO) (eV), energies of lowest unoccupied molecular orbital (LUMO) (eV) and HOMO–LUMO gap (eV) of the most stable Pd_*n*_/coronene (*n* = 2–10) composites calculated at M06-2X/LANL2DZ

Species	S.P	I.P	E.A	HOMO	LUMO	*E*_g_
Coronene	—	6.79	0.90	−6.79	−0.90	5.88
Pd_2_/coronene	Singlet	6.01	1.21	−6.01	−1.21	4.80
Pd_3_/coronene	Singlet	5.95	1.50	−5.95	−1.50	4.45
Pd_4_/coronene	Singlet	5.98	2.24	−5.98	−2.24	3.74
Pd_5_/coronene	Triplet	5.69	1.31	−5.69	−1.31	4.38
Pd_6_/coronene	Triplet	5.75	1.44	−5.75	−1.44	4.30
Pd_7_/coronene	Singlet	5.77	2.21	−5.77	−2.21	3.55
Pd_8_/coronene	Triplet	6.01	2.07	−6.01	−2.07	3.94
Pd_9_/coronene	Triplet	5.88	2.07	−5.88	−2.07	3.82
Pd_10_/coronene	Triplet	5.91	2.20	−5.91	−2.20	3.71

### 3.3 Vertical ionization potential (VIP) and vertical electron affinity (VEA) of the most stable Pd_*n*_/coronene (*n* = 2–10) composites

The change in electronic properties with a change in size of the system can be studied with the help of reactivity descriptors. We have studied a number of reactivity descriptors such as VIP (vertical ionization potential), VEA (vertical electron affinity), chemical hardness, softness, and chemical potential. The main purpose of calculating these properties was to see how the reactivity descriptors change as a function of the size of palladium clusters on coronene. According to Koopmans' theorem, VIPs^52^ of the system can be calculated from the HOMO energy of the neutral systems.^53^ Vertical ionization potential (IP) is the measurement of the amount of energy absorbed when an electron is evacuated from the neutral atom, considering that particle relaxation is not attained during this process.^54^ Vertical ionization potential (VIP) and vertical electron affinity (VEA) are given in Table 2. The VIP value of isolated coronene is 6.79 eV, which decreases to 6.01 eV in the Pd_2_/coronene system. This decrease in the value of VIP is due to the increase in energy of occupied orbitals under the influence of the electron-donating ability of palladium clusters towards coronene. It is obvious from the data that the Pd clusters with an even number of Pd atoms adsorbed on the coronene have greater ionization potential than those with an odd number of palladium atoms, which explains that the system containing an even number of palladium atoms is less reactive than that containing an odd number of palladium atoms. Because of the lowest values of HOMO of Pd_8_/coronene and Pd_2_/coronene, these have the largest value of VIPs (6.01 eV), followed by 5.98 eV for Pd_4_/coronene. These values indicate that a large amount of energy is required to eject the electrons from these systems. Pd_3_/coronene, Pd_4_/coronene, Pd_9_/coronene and Pd_10_/coronene systems have very small difference in values of ionization potential, which are 5.95, 5.98, 5.88 and 5.91 eV, respectively. The lowest value of VIP was indicated in the case of Pd_5_/coronene (5.69 eV). It may be concluded from the above discussion that VIP is lowest when the number of palladium atoms interacting with coronene surface is large.

Electron affinity predicts the stability of a chemical system towards the acceptance of an electron. It gives significant data about the advancement of electronic structure, which depends on size. Electron affinity (EA) is the measurement of the amount of energy liberated when an additional electron is added to a neutral atom, considering particle relaxation is not attained during this process.^55^ Vertical values of electron affinity are given in Table 2. The electron affinity values of our composites increase by the interaction of palladium clusters with coronene. The electron affinity of Pd_2_/coronene complex is 1.21 eV, which is about 0.31 eV higher than the 0.90 eV of coronene itself. VEA values of Pd_2_/coronene and Pd_3_/coronene system are 1.21 eV and 1.50 eV. However, Pd_4_/coronene has the highest value for VEA (2.24 eV). Identical values of VEA for Pd_8_/coronene (2.07 eV) and Pd_9_/coronene (2.07 eV) are noted, which indicate similar stabilities of both systems and the same behaviour toward gaining electrons. Pd_7_/coronene and Pd_10_/coronene exhibit small difference in electron affinity values, *i.e.*, VEA = 2.21 and 2.20 eV for Pd_7_/coronene and Pd_10_/coronene, which illustrates that both systems have the same capability for gaining electrons, with minute difference.

### 3.4 Chemical hardness of the most stable Pd_*n*_/coronene (*n* = 2–10) composites

By utilizing the VIP and VEA values, chemical hardness is determined with the help of eqn (3). Chemical hardness values are given in Table 3. Hardness is the measure of resistance toward charge relocation. Hardness of these systems was indicated through application of the principle of maximum hardness (PMH) given by Pearson. The highest hardness is shown by Pd_2_/coronene (2.40 eV), followed by Pd_3_/coronene (2.23 eV). The results indicate that these systems are quite stable. The hardness of systems decreases with an increase in number of palladium atoms adsorbed on the coronene, but in an irregular way. The lowest hardness is noted for Pd_7_/coronene (1.78 eV), followed by Pd_10_/coronene (1.86 eV) and Pd_4_/coronene (1.87 eV). Chemical hardness values of Pd_8_/coronene (1.97 eV) and Pd_9_/coronene (1.91 eV) are closer to each other. Then, a slight increase in hardness is noted for Pd_5_/coronene and Pd_6_/coronene clusters, *i.e.*, 2.19 and 2.16 eV, respectively. It is obvious from the hardness data that systems with fewer palladium atoms are harder. Less stability is seen for Pd_4_/coronene and Pd_7_/coronene, which showed more reactivity due to a lower value of the chemical hardness.

**Table 3:** Chemical hardness (*η*), softness (*S*), chemical potential (*μ*) and Fermi level (*E*_FL_) of the most stable Pd_*n*_/coronene (*n* = 2–10) composites calculated at M06-2X/LANL2DZ

Composites	Chemical hardness	Softness	Chemical potential	Fermi level
Pd_2_/coronene	2.40	1.04	3.61	−3.61
Pd_3_/coronene	2.23	1.33	3.73	−3.73
Pd_4_/coronene	1.87	2.07	4.11	−4.11
Pd_5_/coronene	2.19	1.13	3.50	−3.50
Pd_6_/coronene	2.16	1.27	3.60	−3.60
Pd_7_/coronene	1.78	2.04	3.99	−3.99
Pd_8_/coronene	1.97	1.90	4.04	−4.04
Pd_9_/coronene	1.91	1.90	3.98	−3.98
Pd_10_/coronene	1.86	2.03	4.06	−4.06

### 3.5 Softness (*S*) of the most stable Pd_*n*_/coronene (*n* = 2–10) composites

Softness of Pd_*n*_/coronene composites is determined by using eqn (4). Polarizability of chemical systems is also related to softness. Softness is the inverse of chemical hardness; therefore, its value also increased regularly with the increase in number of adsorbed palladium atoms on coronene (Table 3). The lowest softness is noted for the Pd_2_/coronene clusters (1.04 eV), which makes it less polarizable. The behaviour of this system is attributed to the much lower number of metal atoms interacting with coronene. A relatively higher value of softness (1.13 eV) was noted for Pd_5_/coronene. The highest softness (2.04 eV) was calculated for Pd_4_/coronene, which points out its high polarizability because of the maximum number of interactions of metals atoms of the clusters with coronene.

Pd_8_/coronene and Pd_9_/coronene clusters exhibit the same softness value (1.90 eV). Pd_7_/coronene has the softness value of 2.04 eV, which is followed by Pd_10_/coronene (2.03 eV). In this study of palladium adsorption on coronene, the overall increasing trend in the values of softness is seen until Pd_4_/coronene, due to the more efficient adsorption of palladium clusters over coronene.

### 3.6 Chemical potential (*μ*) of the most stable Pd_*n*_/coronene (*n* = 2–10) composites

The chemical potential (*μ*) represents the competency of a system towards reactivity. The value of chemical potential^56^ (presented in Table 3) is 3.61 eV in the case of Pd_2_/coronene composite. After the Pd_4_/coronene composite, there is an irregular increasing trend in the chemical potential with the increase in number of palladium atoms in the systems, except for Pd_5_/coronene and Pd_6_/coronene. The highest value of chemical potential is calculated for Pd_4_/coronene (4.11 eV). The lowest chemical potential was observed for Pd_5_/coronene (3.50 eV); therefore, Pd_5_/coronene has less reactivity. The second lowest value of the chemical potential was shown by Pd_6_/coronene (3.60 eV). Pd_9_/coronene and Pd_7_/coronene have almost similar behaviour for chemical potential because there is very minute difference in their chemical potential values (3.98 and 3.99 eV). Pd_8_/coronene has the chemical potential of 4.04 eV. Chemical potentials shown by Pd_3_/coronene and Pd_10_/coronene are 3.73 and 4.06 eV, respectively.

### 3.7 Fermi level (*E*_FL_) of the most stable Pd_*n*_/coronene (*n* = 2–10) composites

At zero Kelvin, Fermi level is the centre of *E*_g_ (HOMO–LUMO energy gap). In the case of the free gas system, *E*_FL_ is indistinguishable from the chemical potential. The values of Fermi level (Table 3) for the Pd_*n*_/coronene (*n* = 2–10) adsorbed system are calculated by using eqn (13). The lowest Fermi level is shown by the Pd_4_/coronene composite, which has a value of −4.11 eV, whereas the highest value of Fermi level is −3.50 eV, which is observed in the case of Pd_5_/coronene. The Fermi level of Pd_2_/coronene, Pd_3_/coronene, Pd_6_/coronene, Pd_7_/coronene, Pd_8_/coronene, Pd_9_/coronene and Pd_10_/coronene systems are −3.61, −3.73, −3.60, −3.99, −4.04, −3.98 and −4.06 eV, respectively. It was noted that a mixed trend of Fermi level is seen in all systems. The systems exhibiting greater values of the Fermi level should have very low energy difference between the highest occupied molecular orbitals (HOMO) and lowest unoccupied molecular orbitals (LUMO), and the appearance of these energy states near the Fermi level results in a lower band gap with the increase in size of the clusters on the coronene.

**Fig. 6:**
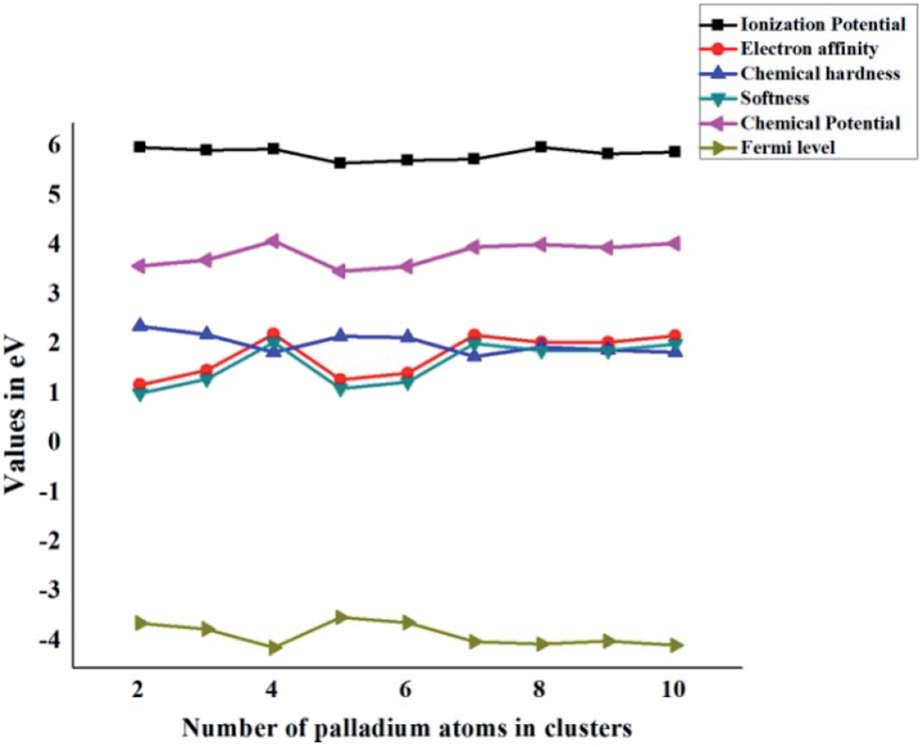
Global chemical indicators (ionization potential, electron affinity, chemical hardness, softness, chemical potential, and Fermi level) of Pd_*n*_/coronene (*n* = 2–10) systems.

All graphs are combined together in Fig. 6. In the case of chemical hardness and Fermi Levels, the minimum value is seen for Pd_7_/coronene and Pd_4_/coronene, whereas the maximum is seen for Pd_2_/coronene and Pd_5_/coronene, respectively. The trends of ionization potential, electron affinities, softness, and chemical potential are almost opposite to those of hardness and Fermi levels. The maximum values of softness, chemical potential, ionization potential and electron affinities are seen for Pd_4_/coronene, Pd_4_/coronene, Pd_2_/coronene and Pd_4_/coronene, whereas the lowest values are seen for Pd_2_/coronene, Pd_5_/coronene, Pd_6_/coronene and Pd_2_/coronene, respectively.

Another interesting aspect revealed from the combined analysis of chemical reactivity descriptors is the behaviour of the Pd_7_/coronene molecule. For example, the lowest hardness (in the entire series) of 1.78 eV is calculated for Pd_7_/coronene. Since hardness depends primarily on the difference between VIP and VEA, these parameters (VEA and VIP) were analyzed. The results clearly reveal that low hardness arises due to high electron affinity and low ionization potential. The vertical electron affinity of Pd_7_/coronene is almost the highest in the series, whereas the vertical ionization potential of this molecule is at the lowest end. The low hardness value reflects its higher reactivity. Since softness is the reciprocal of hardness, Pd_7_/coronene possesses the highest softness. The chemical potential, on the other hand, depends on the sum of VEA and VIP; therefore, the chemical potential does not stand out for Pd_7_/coronene (compared to the rest of the series) mainly due to the cancellation of higher VEA with lower VIP. The chemical potential of Pd_7_/coronene is 3.99 eV, which is comparable to its neighbours in the series.

### 3.8 Molecular electrostatic potential of Pd_*n*_/coronene (*n* = 2–10) composite systems

**Fig. 7:**
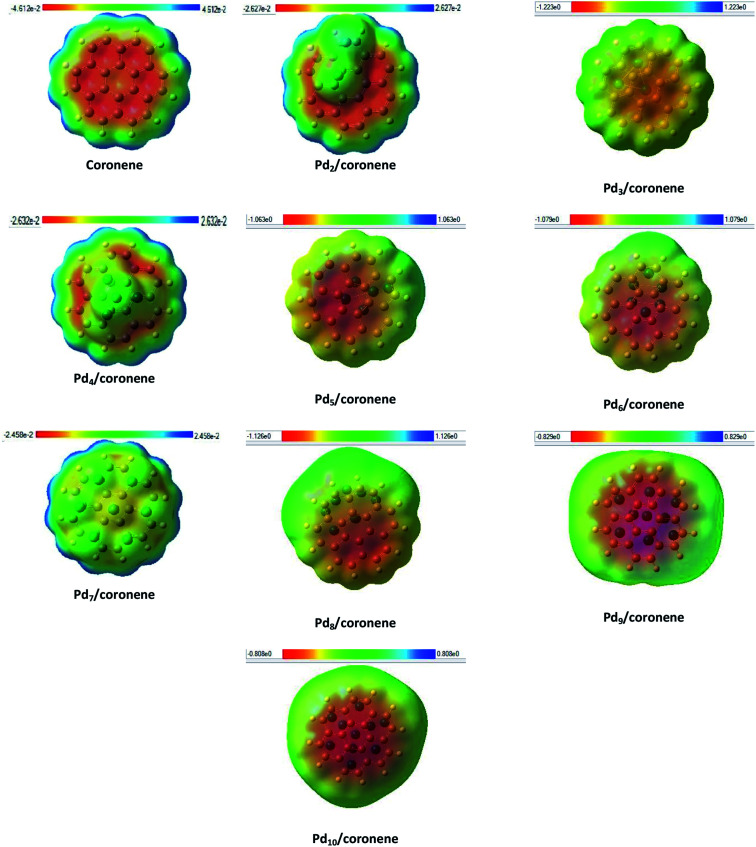
Molecular electrostatic potential (front view) of Pd_*n*_/coronene (*n* = 2–10) composite systems.

Identification of reactive sites can be achieved by molecular electrostatic potential (MEP) surfaces. In MEP surface, potential increases in the order red < orange < yellow < green < blue.^57^ MEP surfaces of our chemical systems are shown in Fig. 7. The MEP diagram of coronene showed that electron deficiencies (blue color) were located on the edges of the coronene, which are more prone to nucleophilic attack, but as we move toward the centre of coronene, the availability of electrons increases and color changes to blue, green, yellow then red at centre of coronene. The red color shows the presence of highly negative potential, which may be due to the delocalization of π electron systems. Adsorption of palladium clusters on coronene resulted in the decrease in negative potential of the coronene. In the case of Pd_2_/coronene, the negative potential decreases on the site where the palladium cluster is adsorbed; however, the areas of electron deficiencies (blue color) of coronene remains unaffected, which is suitable for the electrophilic attack.

The same situation is noticed for the Pd_3_/coronene, Pd_6_/coronene and Pd_8_/coronene systems, but with decreased negative potential. For Pd_7_/coronene system, the adsorption of the palladium clusters on coronene causes unequal distribution of the charges; most of the neutral potential (green color) is located on the palladium atoms of the clusters, and slight negative potential (yellow color) is between these atoms. A very similar nature of charge distribution is shown by the Pd_4_/coronene but with less negative potential when compared with Pd_7_/coronene. In the study of the Pd_*n*_/coronene systems, anomalous charge distribution is shown by Pd_9_/coronene and Pd_10_/coronene, where most of the negative potential lies on the clusters. For all chemical systems, the blue area (electron deficiencies) of coronene remain unaffected, and there is a decrease in negative potential with the increase in number of palladium atoms on coronene because the absorption of clusters on coronene causes distribution of the electronic cloud of coronene away from the absorption site.

### 3.9 NBO charge analysis of Pd_*n*_/coronene (*n* = 2–10) composites

**Fig. 8:**
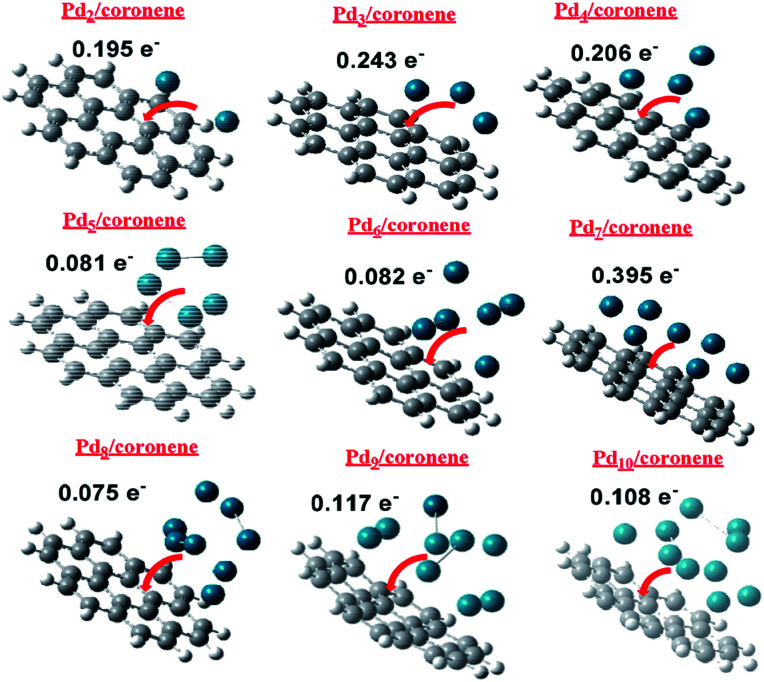
Direction and quantity of charge transfer in Pd_*n*_/coronene (*n* = 2–10).

NBO charge analysis of Pd_*n*_/coronene (*n* = 2–10) was also performed in order to gain understanding about charge transfer upon Pd cluster adsorption on coronene. The direction and amount of charge transfer is shown in Fig. 8. From the figure, it is evident that a mixed trend of charge transfer is seen in composites; the highest charge transfer is noted in Pd_7_/coronene (0.395*e*^−^), which is probably due to the large number of palladium atoms in the low-lying plane, whereas the lowest charge transfer is observed in Pd_8_/coronene (0.075*e*^−^).

The second and third largest values of NBO are noted in Pd_3_/coronene (0.243*e*^−^) and Pd_4_/coronene (0.206*e*^−^), respectively. Overall, good values of charge transfer are seen in all composites upon palladium cluster adsorption on coronene, and all these values suggest that palladium cluster adsorption (on coronene) causes significant charge transfer (Fig. 8) in all systems.

### 3.10 Partial density of states analysis (PDOS) of the most stable Pd_*n*_/coronene (*n* = 2–10) composites

**Fig. 9:**
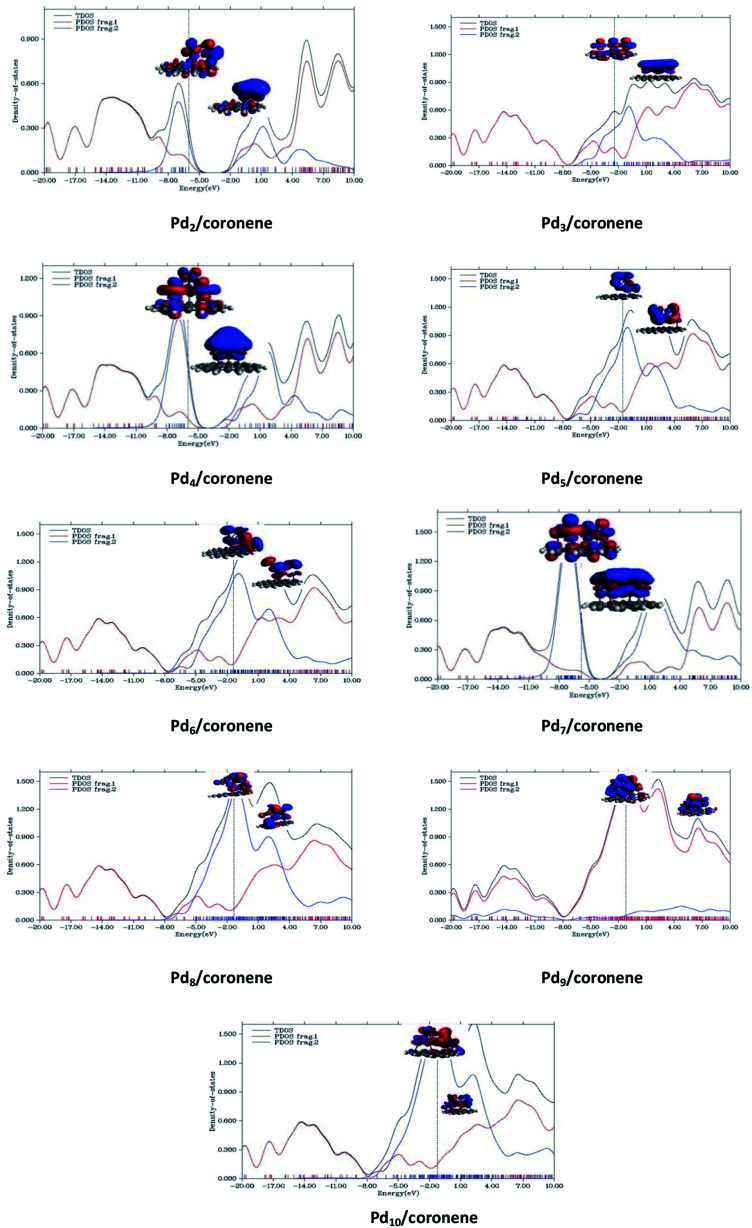
TDOS and PDOS for Pd_*n*_/coronene (*n* = 2–10) composites calculated at M062X/LanL2DZ.

An understanding of the contribution of the different fragments in the frontier molecular orbitals is provided by the partial density of states (PDOS). PDOS for each Pd_*n*_/coronene (*n* = 2–10) composite is shown in Fig. 9, where fragment 1 represents the coronene and fragment 2 represents the metal clusters. The HOMO of all Pd_*n*_/coronene (*n* = 2–10) composites have relatively greater density on the metal cluster than coronene.

## 4. Conclusions

Graphene composites with palladium act as catalyst and adsorption components. We report here the first detailed study on the nature of bonding between graphene (coronene model and palladium Pd_*n*_ clusters (*n* = 2–10). Density functional theory calculations are performed at the M06-2X/LanL2DZ level to analyze the geometric, thermodynamic and electronic properties of palladium–graphene composites. Two different representative models for graphene, coronene and hexabenzocoronene, are used. The adsorption energies calculated at the M06-2X/LANL2DZ level show better agreement with those calculated from MP2/ANO-RCC-VDZP. The adsorption energy analysis reveals that the interaction energies increase with the size of the adsorbed cluster. The difference in behavior between Pd_*n*_/hexabenzocoronene and Pd_*n*_/coronene for interaction energy is attributed to the edge effect present in coronene. The adsorption energy, ionization potential, electron affinity, frontier molecular orbital analysis (HOMO–LUMO), band gap (*E*_g_), chemical hardness (*η*), softness (*S*), chemical potential (*μ*) and Fermi levels (*E*_FL_) are calculated for the most stable palladium coronene composites. Most of the electronic properties show an oscillating behavior when plotted against the number of palladium atoms in the cluster. This oscillating behaviour is due to perturbation of the energies of the frontier orbitals of Pd_5_/coronene and Pd_7_/coronene. The VIP and VEA confirmed that the systems (clusters adsorbed on coronene) are stable in nature with the least reactivity. The HOMO–LUMO gap is dependent on the number of palladium atoms interacting with coronene. The lowest values of HOMO–LUMO gaps are found where favorable interaction exists between coronene and palladium clusters, *i.e.*, Pd_7_/coronene and Pd_10_/coronene, as revealed from the presence of densities on both fragments. MEP analysis shows that the increase in cluster size adsorbed on the coronene causes greater transfer of negative charge from the clusters to coronene. The same trend is noted for the adsorption energy, where there is an increase in adsorption energy with an increase in the number of palladium atoms on coronene, which shows the strong adsorption of clusters having a greater number of palladium atoms. The outcome of the present study is the improved electronic properties of the palladium cluster-based graphene system, which can confidently be used for sensing purposes.

## Conflicts of interest

No conflicts declared.

## Supplementary Material
